# The *Shiny Balancer* - software and imbalance criteria for optimally balanced treatment allocation in small RCTs and cRCTs

**DOI:** 10.1186/s12874-018-0551-5

**Published:** 2018-10-16

**Authors:** Thomas Grischott

**Affiliations:** Institute of Primary Care, University and University Hospital of Zurich, Pestalozzistrasse 24, CH-8091 Zurich, Switzerland

**Keywords:** Randomisation, Minimisation, Treatment allocation, Covariate-adaptive, Imbalance measure, Imbalance metric, Cluster randomised trial

## Abstract

**Background:**

In randomised controlled trials with only few randomisation units, treatment allocation may be challenging if balanced distributions of many covariates or baseline outcome measures are desired across all treatment groups. Both traditional approaches, stratified randomisation and allocation by minimisation, have their own limitations. A third method for achieving balance consists of randomly choosing from a preselected list of sufficiently balanced allocations. As with minimisation, this method requires that heterogeneity between treatment groups is measured by specified imbalance metrics. Although certain imbalance measures are more commonly used than others, to the author's knowledge there is no generally accepted “gold standard”, neither for categorical and even less so for continuous variables.

**Methods:**

An intuitive and easily accessible web-based software tool was developed which allows for balancing multiple variables of different types and using various imbalance metrics. Different metrics were compared in a simulation study.

**Results:**

Using simulated data, it could be shown that for categorical variables, *χ*^2^-based imbalance measures seem to be viable alternatives to the established “quadratic imbalance” metric. For continuous variables, using the area between the empirical cumulative distribution functions or the largest difference in the three pairs of quartiles is recommended to measure imbalance. Another imbalance metric suggested in the literature for continuous variables, the (symmetrised) Kullback-Leibler divergence, should be used with caution.

**Conclusion:**

The *Shiny Balancer* offers the possibility to visually explore the balancing properties of several well established or newly suggested imbalance metrics, and its use is particularly advocated in clinical studies with few randomisation units, as it is typically the case in cluster randomised trials.

**Electronic supplementary material:**

The online version of this article (10.1186/s12874-018-0551-5) contains supplementary material, which is available to authorized users.

## Background

Small numbers of subjects combined with large numbers of covariates may pose particular challenges for treatment allocation in randomised controlled trials (RCTs) since simple randomisation does not necessarily ensure balance, i.e. similar distributions of all important covariates, across all treatment groups. The same difficulty may arise when balance is required with respect to baseline outcome measures collected before randomisation. Situations with few allocation units can occur both in trials with individually randomised designs, e.g. if interim analyses are planned with only part of the subjects, or – perhaps even more commonly – with cluster designs, e.g. in Primary Care research, where cluster-RCTs with relatively few clusters but many potential prognostic factors are a popular study design. In this latter case of cluster (or group) randomised trials, balance may be desired for cluster-level attributes as well as for cluster-level aggregates of individual characteristics.

A recent overview of alternative randomisation methods to achieve balance over all treatment arms, together with their merits and disadvantages, can be found in [1]. The two classic methods are stratified randomisation and minimisation [2, 3]. For stratified randomisation, all variables need to be categorised, and randomised allocation is then performed separately within all combinations of such categories, the so-called strata. Unfortunately, even small numbers of variables lead to rather large numbers of sparsely populated strata, thus drastically limiting the applicability of this method.

Minimisation, on the other hand, involves the definition of imbalance measures for each variable (or type of variable). Total imbalance of an allocation scheme is usually defined to be the (unweighted or weighted) sum of the imbalance contributions resulting from all variables [4, 5]. The randomisation units are sequentially allocated to the treatment group where their allocation leads to a smaller total imbalance. This allocation to the “better” treatment can be done purely deterministically or with a certain probability *p* > 50%. Choosing *p* close to 1 results in high balance at the cost of “randomness”, and in the extreme case of deterministic allocation, the trial cannot rightly be called a “randomised” trial anymore. Low values of *p* preserve much randomness in the allocation procedure, thus minimising the possibility of selection bias, but may lead to imbalanced allocation schemes, similar to simple randomisation. Due to its sequential nature, minimisation is more likely to be used in trials with randomisation of sequentially included individuals, rather than in cluster randomised designs where all participating clusters are often identified pre-randomisation.

A third method has been proposed by Raab and Butcher in 2001 [6], by Moulton in 2004 [7], by Carter et al. in 2008 [8], and again under the name “studywise minimization” by Perry et al. in 2010 [9]. A slightly modified approach, the “best balance” allocation method, was recently presented by de Hoop et al. [10]. According to these authors, the method is applicable if all study subjects (units) with all their covariate values are known at the time of allocation. First, the full set of all possible allocation schemes is constructed, or, in case this is computationally not feasible, a random subset of the full set may be sampled instead. In the second step, for each of the generated allocation schemes its total imbalance is calculated. Next, a specified number or proportion of sufficiently balanced allocation schemes is preselected. In the fourth and last step, the researcher – or an independent second party – choses the final allocation scheme at random among those identified in the previous step.

An obvious advantage of this third approach is its superior ability to achieve balance even with regard to a large number of variables. This has been shown in simulation studies [9, 11], but in fact, is inherent in the design of the method. The researcher can not only be sure that her/his final allocation scheme respects the specified total imbalance level, but is also offered insight into what range of imbalances is at all achievable. A somewhat more subtle advantage lies in the fact that preserving a controlled amount of randomness has desirable implications for the later statistical analysis [12]. An in-depth evaluation of the method for the design and analysis of cluster randomised trials can be found in two recent articles by Li et al. [13, 14].

Despite its advantages, the method has not gained much popularity yet [15]. This might be due to the lack of a platform independent, intuitive and easy-to-use and at the same time highly customisable software solution. Therefore, the first aim of the present paper is to fill this gap by providing the web-based allocation tool *Shiny Balancer* and to demonstrate its capabilities which partly exceed those of similar existing tools. Possible uses cover balancing with regard to multiple discrete or continuous covariates or baseline outcome measures in individually randomised trials as well as cluster attributes or cluster-level aggregates of individual variables in cluster randomised trials. The *Shiny Balancer* can be accessed through any web browser, or (customised and) run locally on any computer with RStudio [16] installed.

Historically, allocation by minimisation was first introduced for categorical covariates. Taves [17] used the sum (over all covariate levels) of absolute differences in numbers between the two treatment groups as his original imbalance measure. (This is equivalent to the “average imbalance” in the terminology of Perry et al. [9].) Pocock and Simon [18] introduced the sum of the squared differences as an alternative imbalance measure (called “quadratic imbalance” by Perry et al. and others). Even though other imbalance measures have been proposed [9, 19], Pocock and Simon’s “quadratic imbalance” has become established as the de-facto standard in minimising imbalance from categorical covariates. It is sometimes also called the “variance method of minimization” [9]. However, there are still other conceivable and potentially promising imbalance metrics which have neither been suggested nor studied yet.

More recently, several attempts emerged in the literature to balance both categorical and continuous covariates within a comprehensive framework [19]. Among the suggested imbalance measures for continuous covariates are the *p*-value of the Wilcoxon U test [20], the largest difference of the three pairs of quartiles [21], the area between the two empirical cumulative distribution functions [22], and the Kullback-Leibler divergence [23]. For a recent overview of proposed imbalance measures see [19]. To the author’s knowledge, there is no general consensus among experts as to which of these measures is preferable.

The second aim of this paper is therefore to compare the established imbalance metrics for categorical variables among themselves and also with several newly suggested ones, and to do the same with the imbalance measures for continuous variables, again introducing new suggestions. Comparisons will be done using the source code of the *Shiny Balancer* with two simulated sets of allocation units with matching covariables.

## Implementation

### The *Shiny Balancer*

The *Shiny Balancer* can be accessed via its URL [24] using any web browser. Alternatively, the source code can be downloaded (Additional file 1: app.R and helpers.R) and run on any computer with RStudio installed. In the latter case, the following four standard libraries need to be loaded: *shiny*, *rhandsontable*, *xlsx*, and *flexmix*. The first run of the software will tell if any of them are missing.

**Fig. 1:**
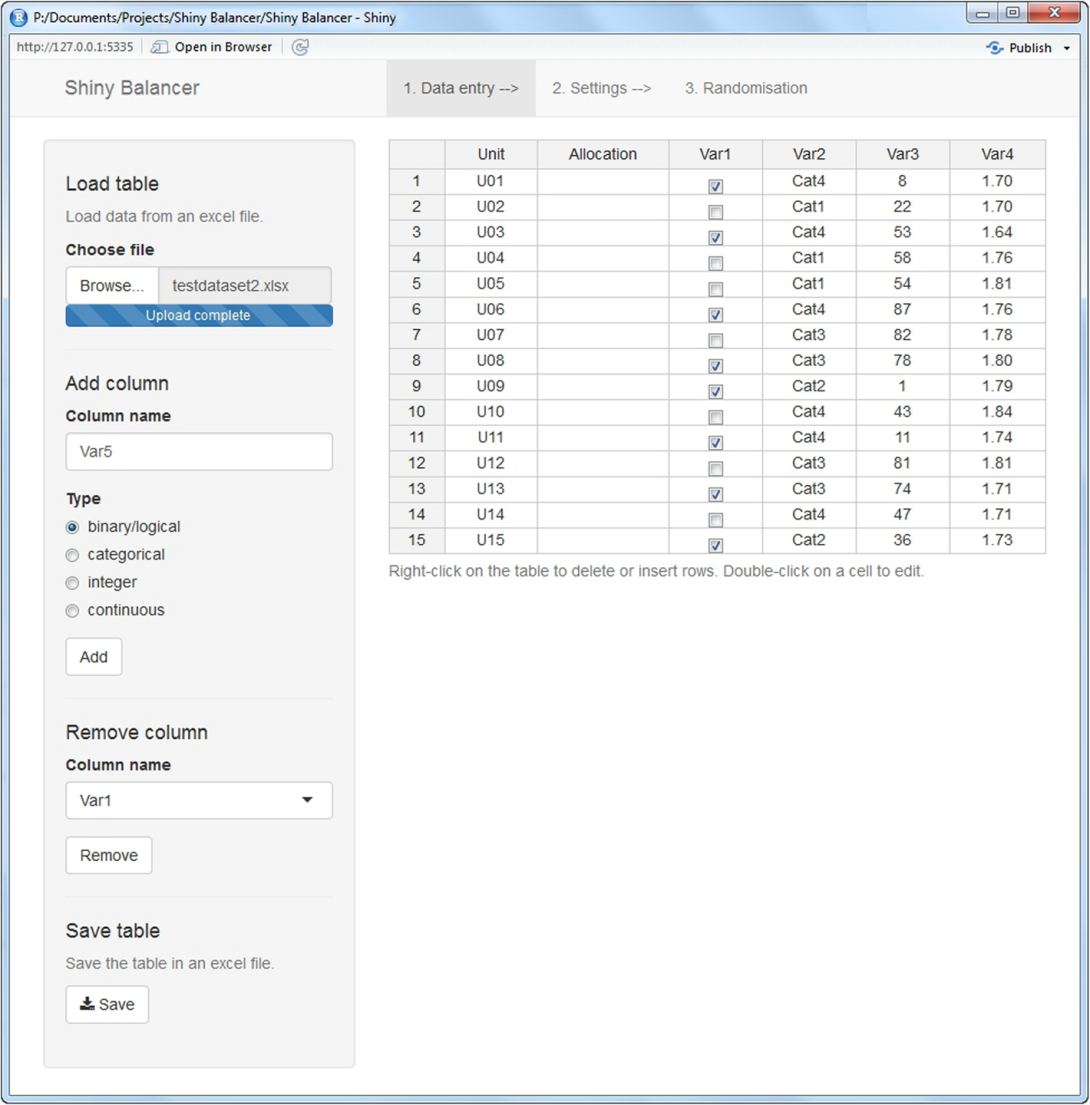
The data entry tab of the *Shiny Balancer*

Figure 1 shows the data input screen as the first of three tabs of the *Shiny Balancer*. The adaptation of the data table either manually or by importing a pre-existing excel file is mostly self-explaining. The second column (*Allocation*) may contain treatment allocations that have already been assigned previously. In this case, treatment groups must be coded by A or B, respectively.

**Fig. 2:**
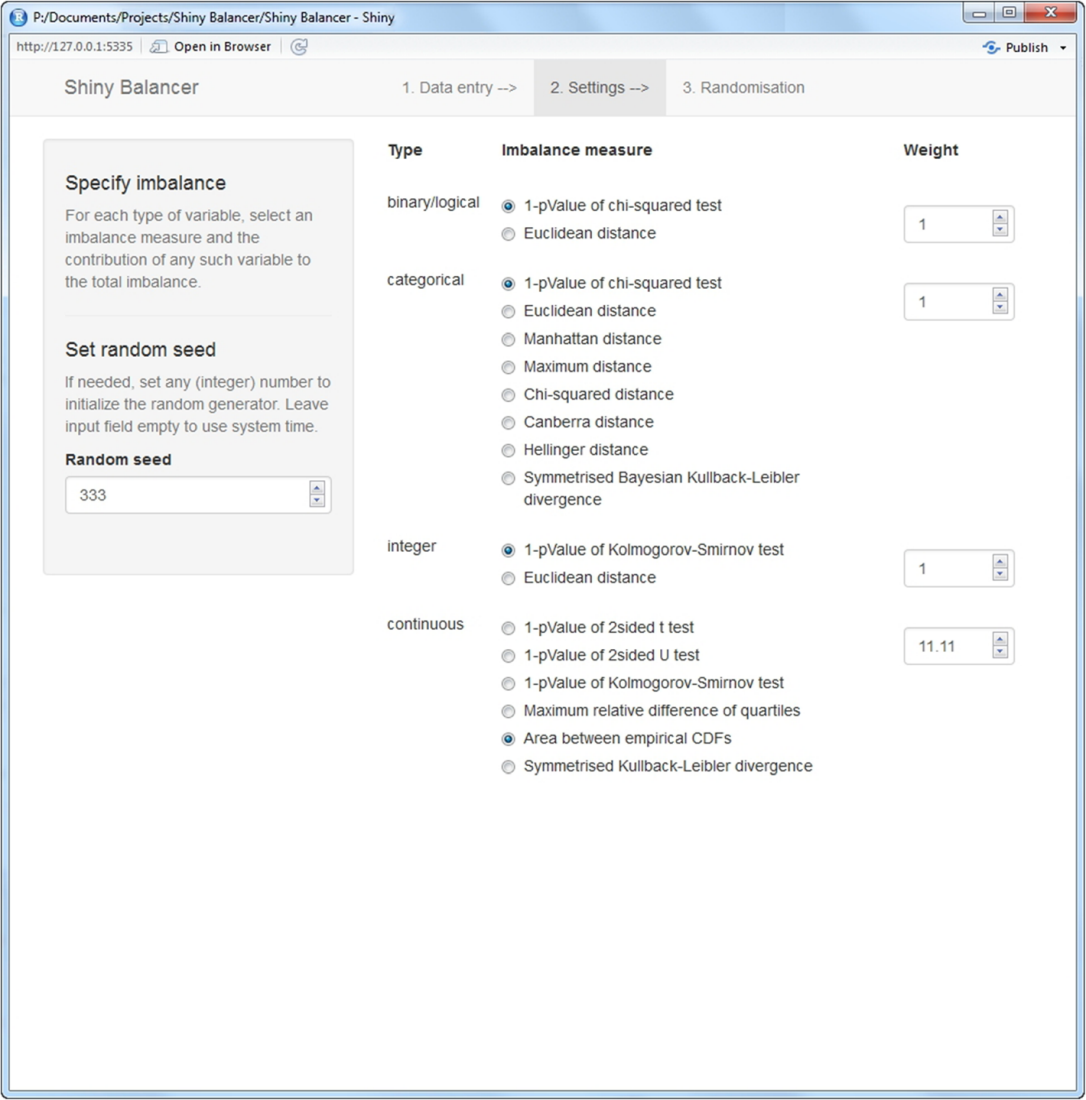
The settings tab of the *Shiny Balancer*

Figure 2 shows the settings panel. For each type of variable, a suitable imbalance metric together with the weight of the respective variable(s) in the total imbalance can be selected. Definitions of the various imbalance measures will be given below in the next section. By setting the weight of a specific variable type to the inverse of the maximum imbalance as calculated in a previous run (with all other weights set to 0), the range of imbalance contributions from variables of that specific type can be standardised to the interval from 0 to 1. For reproducibility, a random seed may be set to initialise the random generator.

**Fig. 3:**
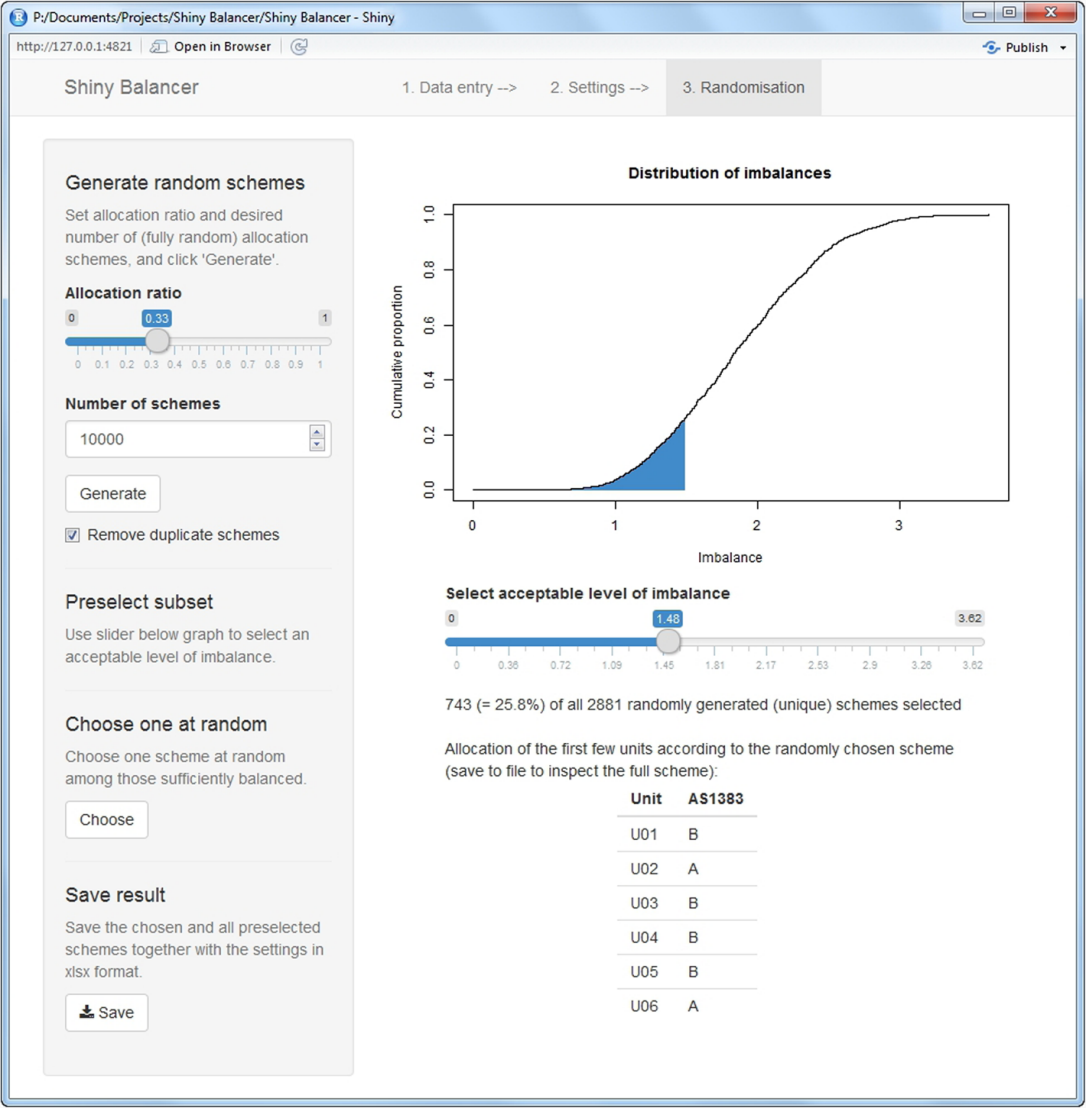
The result of a completed allocation procedure shown in the randomisation tab of the *Shiny Balancer*

Figure 3 shows the randomisation tab of the *Shiny Balancer*. First, the relative size of treatment arm A needs to be specified using the *Allocation ratio* slider. Pressing the *Generate* button then creates the desired *Number of schemes* by repeatedly shuffling a vector with As and Bs in the correct proportions. Since this might result in the same allocation scheme generated more than once, duplicate schemes can be removed by selecting the designated tick box. After the required number of unconstrained random allocation schemes with their respective imbalances have been calculated, their cumulative distribution function is plotted. Using the slider below the plotting area, a set of sufficiently balanced allocation schemes can be preselected, either via their number, proportion or maximum imbalance value.

Once the set of random allocation schemes has been generated and a subset of acceptably balanced schemes preselected, this subset may be saved using the *Save* button in the bottom left corner. An independent second party (or some defined external random mechanism) can then choose the final allocation scheme at random among those preselected. Alternatively, the *Shiny Balancer* carries out this last step itself if the *Choose* button is pressed. The allocation of the first 6 units is shown together with the identifying number of the final allocation scheme in the program interface, and the full final scheme can be exported using the same *Save* button mentioned above.

### Imbalance measures

For a specific allocation scheme with one single binary, categorical or integer variable, let ${n}_x^A$ and ${p}_x^A$ be the number and proportion of units allocated to treatment A which fall into category *x*, and assume ${n}_x^B$ and ${p}_x^B$ to be defined similarly. Then:

1-PX2: The first imbalance metric, *1-pValue of chi-squared test*, is defined in the obvious way via a 2 × *k*-contingency table containing all ${n}_x^A$ and ${n}_x^B$ (where *k* is the number of levels). The *p*-value was used (instead of the test statistic itself) to achieve an imbalance range from 0 to 1, and had to be subtracted from 1 to be increasing with decreasing balance.
Eucl: The *Euclidean distance* $\sqrt{\sum_x{\left({p}_x^A-{p}_x^B\right)}^2}$corresponds to the “quadratic imbalance” in Perry et al.’s terminology [9], and to the “BB score” in de Hoop et al. [10] and Li et al. [13].
Manh: The *Manhattan distance*${\sum}_x\ \left|{p}_x^A-{p}_x^B\right|$corresponds to the “average imbalance” in Perry’s terminology and to the “range imbalance” in Zielhuis et al. [25].
Max: The *Maximum distance* ${\max}_x\ \left|{p}_x^A-{p}_x^B\right|$is similar to the “maximal imbalance” by Perry et al. and to the “TB score” according to Li’s terminology.
X2d: The *Chi-squared distance*, here defined as$\sqrt{\ {\sum}_x\frac{{\left({p}_x^A-{p}_x^B\right)}^2}{p_x^A+{p}_x^B}}$, is a standardised version of the Euclidean distance.
Canb: The *Canberra distance* ${\sum}_x\ \frac{\left|{p}_x^A-{p}_x^B\right|}{p_x^A+{p}_x^B}$is termed “standardized range” by Zielhuis.
Hell: The *Hellinger distance* is defined as$\sqrt{1-{\sum}_x\sqrt{p_x^A\cdot {p}_x^B}}$.
SBKL: The somewhat freely named *Symmetrised Bayesian Kullback-Leibler divergence* is defined as ${\sum}_x{\overset{\sim }{p}}_x^A\cdot \ln \frac{{\overset{\sim }{p}}_x^A}{{\overset{\sim }{p}}_x^B}+{\sum}_x{\overset{\sim }{p}}_x^B\cdot \ln \frac{{\overset{\sim }{p}}_x^B}{{\overset{\sim }{p}}_x^A},$where ${\overset{\sim }{p}}_x^{A,B}={p}_x^{A,B}\left({n}_x^{A,B}+1\right)$ to ensure non-zero denominators.
1-PKS: The *1-pValue of Kolmogorov-Smirnov test* is again defined in the obvious way via the maximal distance between the empirical distribution functions of the two samples in treatment groups A and B, respectively.

For continuous variables, the imbalance measures are defined as follows:

1-Pt: *1-pValue of 2sided t test* is defined in the obvious way.
1-PU: *1-pValue of 2sided* U *test* is – also rather obviously – defined as 1 minus the *p*-value of the Mann-Whitney U test for two independent samples. This imbalance metric has been proposed by Frane [20].
1-PKS: *1-pValue of Kolmogorov-Smirnov test* has already been explained.
Mrdq: For the *Maximum relative difference of quartiles*, in both treatment groups the lower quartiles ${q}_{0.25}^A$ and ${q}_{0.25}^B$, the two medians *m*^*A*^ and *m*^*B*^, and the upper quartiles ${q}_{0.75}^A$ and ${q}_{0.75}^B$ are calculated. The imbalance contribution from the continuous variable is then given by $\max\ \left\{\frac{\left|{q}_{0.25}^A-{q}_{0.25}^B\right|}{\max \left\{\left|{q}_{0.25}^A\right|,\kern0.5em \left|{q}_{0.25}^B\right|\right\}},\frac{\left|{m}^A-{m}^B\right|}{\max \left\{\left|{m}^A\right|,\kern0.5em \left|{m}^B\right|\right\}},\frac{\left|{q}_{0.75}^A-{q}_{0.75}^B\right|}{\max \left\{\left|{q}_{0.75}^A\right|,\kern0.5em \left|{q}_{0.75}^B\right|\right\}}\ \right\}$. This measure corresponds to a suggestion by Su [21].
AbCDF: The self-explaining *Area between empirical CDFs* has been proposed as a measure of imbalance by Lin and Su [22].
SKL: The *Symmetrised Kullback-Leibler divergence* – as proposed by Endo et al. [23] – is calculated by estimating density functions *p*^*A*^(*x*) and *p*^*B*^(*x*) of the samples in both treatment groups, followed by numerical integration: $\int {p}^A(x)\cdot \ln \frac{p^A(x)}{p^B(x)} dx+\int {p}^B(x)\cdot \ln \frac{p^B(x)}{p^A(x)} dx$

All imbalance measures can easily be modified or customised in the source code (e.g. if the use of ${n}_x^A$ and ${n}_x^B$ is preferred over ${p}_x^A$ and ${p}_x^B$, or if different standardisations are to be used). Moreover, the source code contains a template imbalance function for straightforward inclusion of new imbalance metrics.

### Simulation

In order to compare different imbalance measures, two sets of test data were simulated, each containing four variables, i.e. one of each type. Data set 1 includes 14 randomisation units to be allocated to equally sized treatment arms. Data set 2 consists of 15 units to be allocated at a ratio of one third to two thirds. Both the R code used to generate the test data and the data sets themselves can be found in the additional files section (Additional file 1: simulations.R, testdataset1.xlsx, and testdataset2.xlsx).

For both sets of test data, 10′000 allocation schemes were generated. After excluding duplicate schemes, 3′246 and 2′881 unique schemes remained, corresponding to 3′246/3′432 = 94.6% and 2′881/3′003 = 95.9% of all possible allocation schemes, respectively, which was considered sufficient coverage.

For each variable in both data sets, its standardised contributions to the total imbalances were calculated applying all type-matching imbalance measures to every unique allocation scheme. Imbalance contributions according to different imbalance measures were plotted and compared pairwise using Spearman’s rank correlation *ρ*_*S*_.

Finally, to demonstrate the performance of the *Shiny Balancer* when balancing jointly on more than one variable, the 10 and 100 “best” allocation schemes (i.e. those with the smallest total imbalance resulting from standardised contributions of all variables) were calculated for both data sets and specifically selected quadruples of imbalance measures. These best allocation schemes, together with all schemes generated, were then plotted as quadrangles in four-spoked radar charts, with the spokes representing the standardised imbalance contributions of the individual variables.

## Results

The full results of the simulation study can be found online (Additional file 1: results_xxx_tdsy.jpg, where xxx denotes the variable type and y is the number of the data set).

Both of the two imbalance metrics offered by the *Shiny Balancer* for binary variables, 1-PX2 and Eucl (Fig. 2), lead to the same subset of allocation schemes with minimal imbalance contribution. This optimal subset consists of roughly 40% of all unique allocation schemes (41.4% in data set 1, 39.2% in data set 2). Moreover, the imbalance contributions according to both metrics can take the same number of different values (4 different values in data set 1, 6 different values in data set 2). Spearman’s rank correlation coefficient *ρ*_*S*_ between the imbalance contributions of the two metrics is 1 (in both data sets), meaning that both imbalance metrics rank all allocation schemes in exactly the same order (Additional file 1: results_bin_tds1.jpg and results_bin_tds2.jpg).

**Fig. 4:**
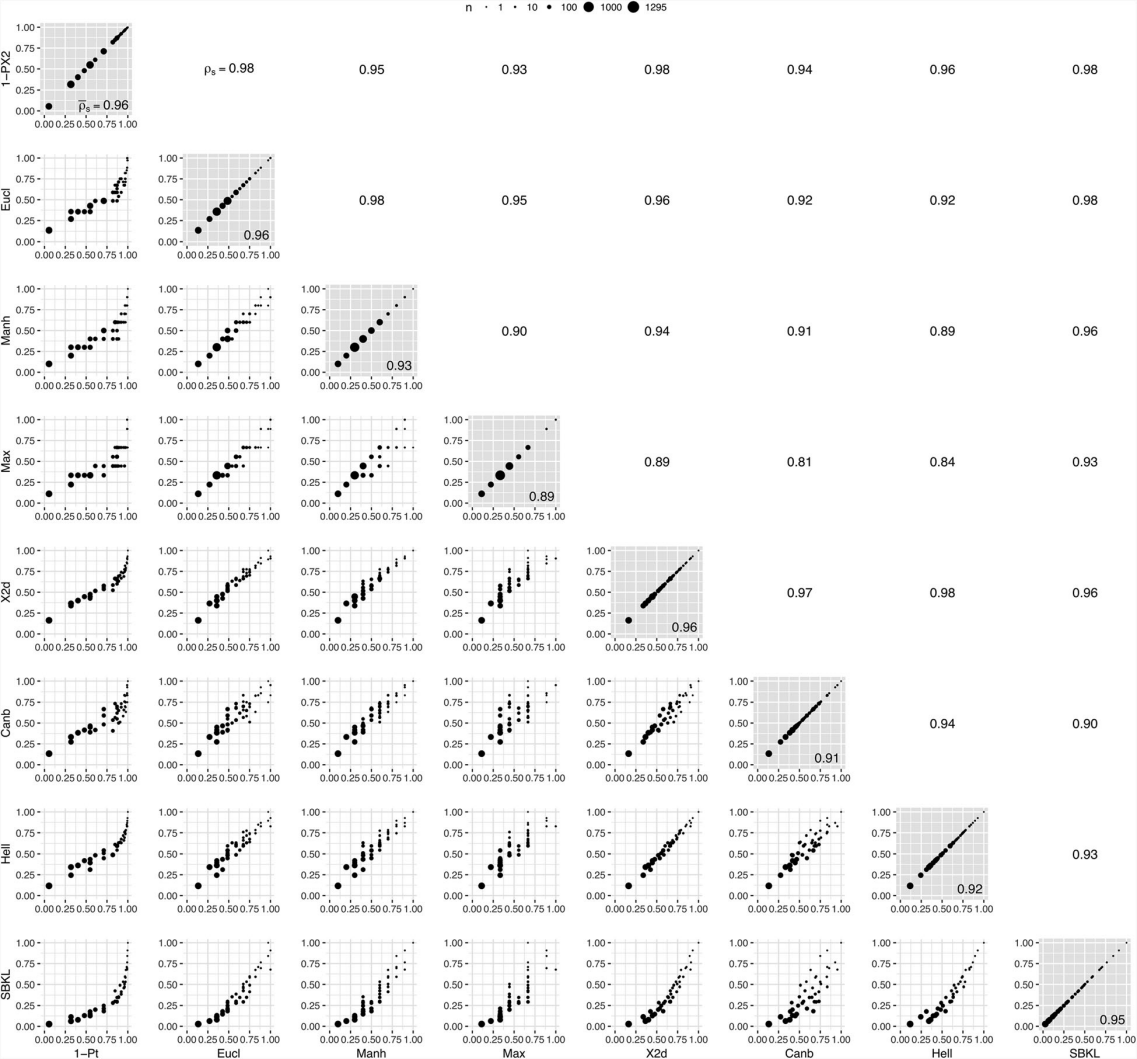
Pairwise comparison of imbalance metrics for categorical variables using data set 2 (*n*: number of allocation schemes; *ρ*_*S*_: Spearman’s rank correlation coefficient; ${\overline{\rho}}_S$: mean rank correlation coefficient)

The situation gets somewhat more interesting with categorical variables. Again, all (eight) imbalance metrics implemented, 1-PX2, Eucl, Manh, Max, X2d, Canb, Hell, and SKBL (Fig. 2), lead to the same subset of allocation schemes with minimal imbalance contribution. This optimal subset includes 23.4% of all unique allocation schemes simulated for data set 1, and 12.0% in the case of data set 2. The number of different values which the imbalance contributions can take differs between different metrics, with the number being highest for the *Symmetrised Bayesian Kullback-Leibler divergence* (SKLD) and the *Hellinger distance* (Hell) (7 and 7 different values with data set 1, 41 and 34 different values with data set 2) and lowest for the *Manhattan* (Manh) and *Maximum* (Max) *distances* (4 and 4 with data set 1, 10 and 8 with data set 2). The highest average rank correlation ${\overline{\rho}}_S$ of one specific imbalance measure with all others can be observed for the *1-pValue of chi-squared test* (1-PX2) metric (0.98 in data set 1, 0.96 in data set 2), followed by the *Chi-squared distance* (X2d) (0.96 in both data sets). At the other end of the scale, the *Maximum distance* correlates relatively poorly with its competing metrics (${\overline{\rho}}_S$ = 0.93 in data set 1, and 0.89 in data set 2). From all *ρ*_*S*_ < 1 in data set 2, it can be concluded that no two measures rank all the allocation schemes exactly in the same order, but most discrepancies occur only at relatively high imbalance values (Additional file 1: results_cat_tds1.jpg; Fig. 4).

**Fig. 5:**
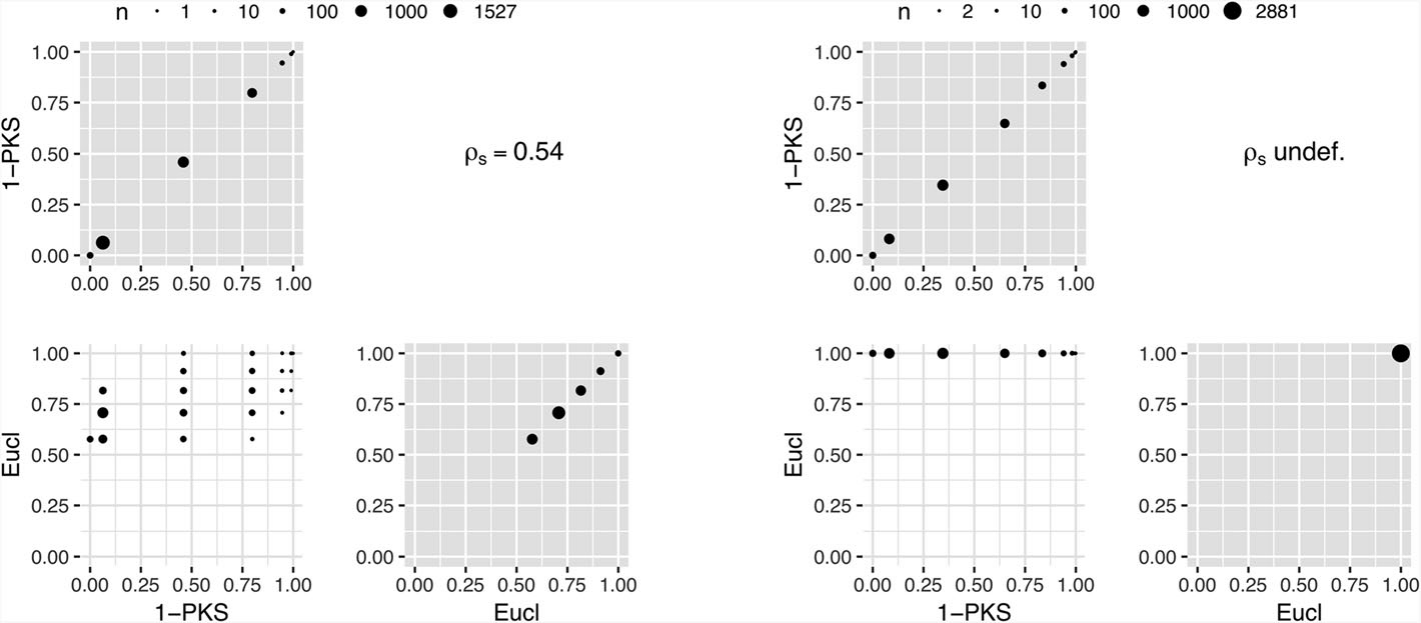
Comparison of imbalance metrics for integer variables (left: data set 1; right: data set 2; *n*: number of allocation schemes; *ρ*_*S*_: Spearman’s rank correlation coefficient)

Within the present context, integer type variables may be treated in a similar manner to either categorical or continuous variables, i.e. their imbalance contributions can be calculated using either measures for categorical or for continuous variables. The two choices offered by the *Shiny Balancer*, 1-PKS and Eucl (Fig. 2), stand as examples for the two larger classes of possible imbalance metrics. Measures for categorical variables must be used with particular caution in situations with small numbers of mostly different observed values, since this will lead to sparsely populated categories. This can be observed nicely in data set 2, where using the *Euclidean distance* (Eucl) results in exactly the same imbalance contributions from all 2′881 (unique) allocation schemes (Fig. 5).

**Fig. 6:**
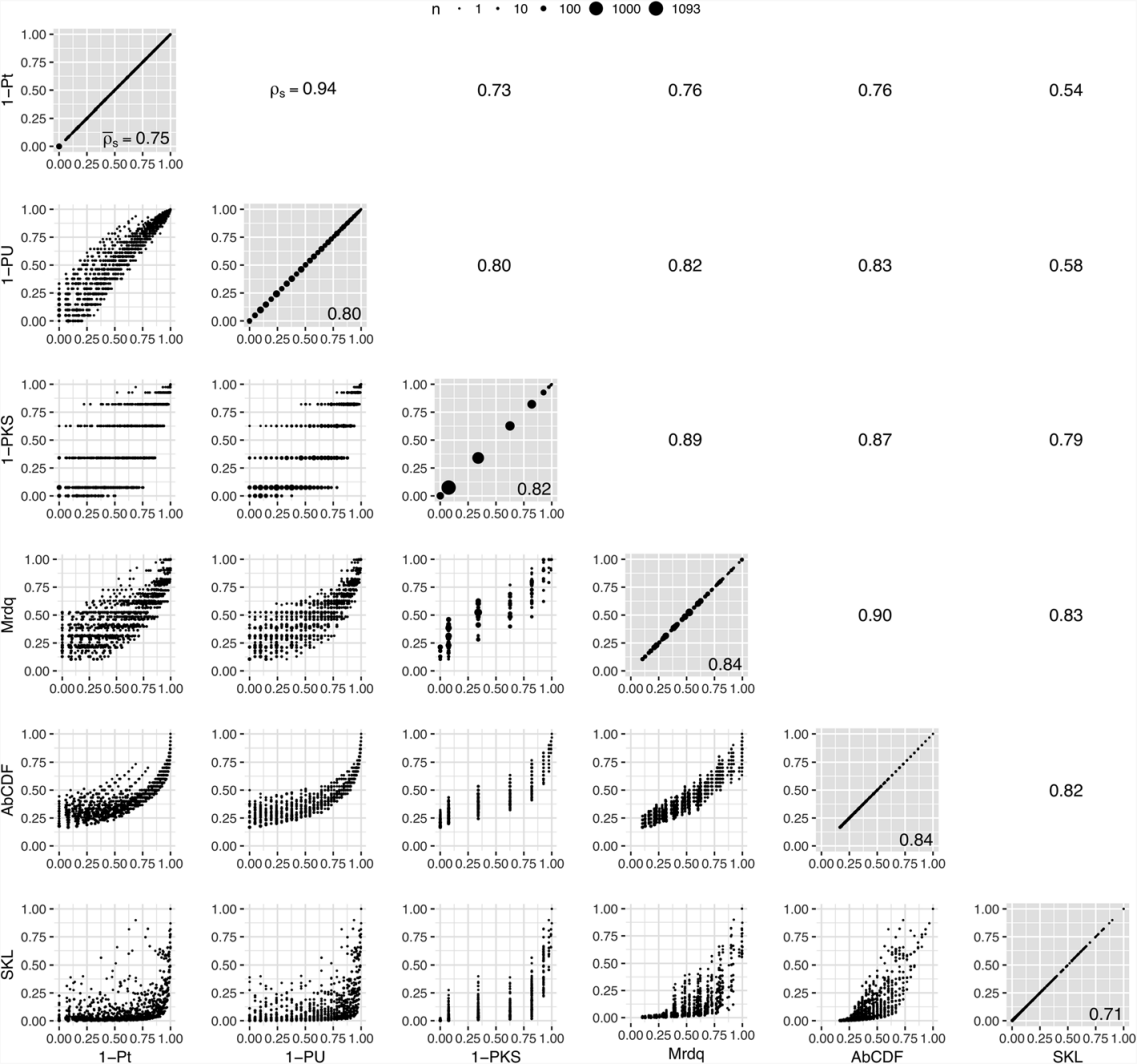
Pairwise comparison of imbalance metrics for continuous variables using data set 2 (*n*: number of allocation schemes; *ρ*_*S*_: Spearman’s rank correlation coefficient; ${\overline{\rho}}_S$: mean rank correlation coefficient)

Contrary to the other variable types, in the case of continuous variables different imbalance measures do not always lead to the same subset of “most balanced” allocation schemes. As can be seen in Fig. 6, there are, for example, allocation schemes which optimally balance the continuous variable according to the imbalance metric *1-pValue of 2sided t test* (1-Pt) but not according to the *Maximum relative difference of quartiles* (Mrdq) or the *Area between empirical CDFs* (AbCDF) criteria. This goes hand-in-hand with smaller rank correlations (from 0.44 to 0.89 in data set 1 and from 0.54 to 0.94 in data set 2). The two imbalance measures which – consistently over both data sets – correlate best with all other measures, are the *Area between empirical CDFs* (${\overline{\rho}}_S$ = 0.81 in data set 1, and 0.84 in data set 2) and the *Maximum relative difference of quartiles* (${\overline{\rho}}_S$ = 0.77 in data set 1, and again 0.84 in data set 2), while the metric which correlates least with all others is the *Symmetrised Kullback-Leibler divergence* (SKL) (${\overline{\rho}}_S$ = 0.67 in data set 1, and 0.71 in data set 2). Continuous variables with near optimal balance according to this metric can be almost anything from perfectly balanced to surprisingly imbalanced with respect to other imbalance measures (Additional file 1: results_con_tds1.jpg and results_con_tds2.jpg). By contrast not surprisingly at all, the number of distinct values which the imbalance contributions can obtain is larger than in the case of categorical variables. However, this number is relatively small, compared to other metrics, for *1-pValue of 2sided* U *test* (1-PU) (25 different values with data set 1, 49 different values with data set 2), for *1-pValue of Kolmogorov-Smirnov test* (1-PKS) (7 different values with data set 1, 10 different values with data set 2), and for *Maximum relative difference of quartiles* (72 different values with data set 1, 62 different values with data set 2).

**Fig. 7:**
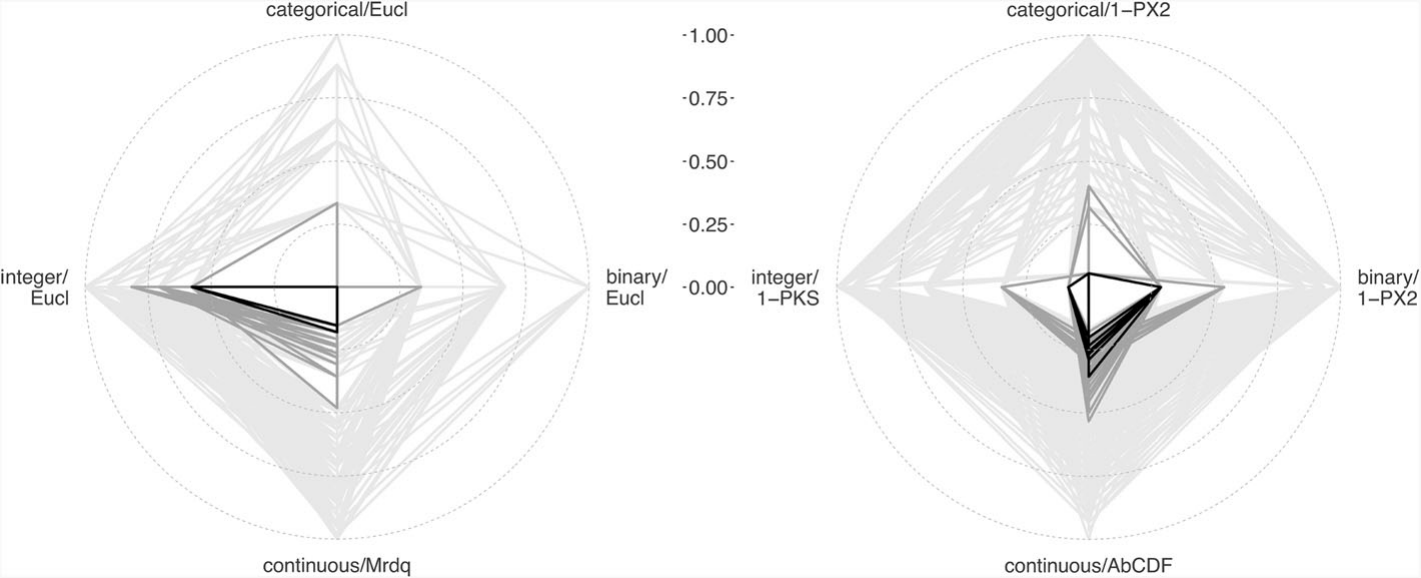
Balancing on multiple variables in data set 1 (left) and data set 2 (right). Allocation schemes are represented by quadrangles whose vertices indicate the standardised imbalance contributions from individual variables of given types according to the specified metrics. Black: 10 schemes with smallest total imbalance; dark grey: 90 “next best” schemes; light grey: all generated schemes

Figure 7 shows the performance of the *Shiny Balancer* in balancing on all four variables in the two simulated data sets. The imbalance contributions of each single variable in optimal and almost optimal allocation schemes can be compared to the minimum imbalance achievable when balancing on the respective variable alone.

## Discussion

While some imbalance measures like the “quadratic imbalance” are preferably used in minimisation algorithms, to the author’s knowledge no measure has been generally accepted as the “gold standard”, neither for categorical variables and even less so for continuous variables. In the present paper, comparisons are therefore not made to some reference method but within a set of several established or newly introduced imbalance measures. A strong rank correlation with other measures and a high number of possible values (allowing for a finer gradation when preselecting a set of sufficiently balanced schemes) are considered necessary (but certainly not sufficient) criteria for an acceptable imbalance metric. Of course, this approach incurs the (theoretical) risk of wrongfully discredit an imbalance metric which could in truth be the only one acceptable among several inappropriate but highly correlated competitors.

In general and in addition to the recommendations following below, it is suggested to experiment with different imbalance measures for each individual variable. (This can be achieved by setting all other weights to 0.) The range of the imbalance contributions from a specific variable can be standardised by setting its weight to the inverse of the maximum imbalance contribution obtained from a previous simulation. Standardised imbalance distributions should then be assessed visually with regard to the number (i.e. the number of different imbalance values) and heights (i.e. the number of allocation schemes with the same imbalance value) of the steps in the cumulative distribution function. When choosing the appropriate imbalance metric, the statistical methods to be used in the later analyses may also be taken into account. (If, for example, outcome measures will be compared using *χ*^2^-tests then *χ*^2^-based metrics seem to be natural choices to asses baseline imbalance.)

For binary variables, all implemented imbalance measures are equivalent. When there is no specific reason for another choice, the use of *1-pValue of chi-squared test* is recommended because of its conceptual familiarity and its intrinsic standardisation. This choice is also in accordance with the next recommendation.

For categorical variables, both *1-pValue of chi-squared test* and the *Chi-squared distanc*e seem to be advisable options. Both measures correlate highly with the other measures, and the numbers of different values which the imbalance contributions from these two measures can take are still relatively high. However, if only the most balanced allocation schemes shall be considered for preselection, then all implemented metrics may be used. (Note that, contrary to the strategy presented here, the usual minimisation algorithm does not guarantee that the imbalance stays within controllable limits. At least in theory, an imbalance sequence obtained from sequential allocation by (stochastic) minimisation may instead reach levels where the choice of imbalance metric does in fact matter.)

When choosing the imbalance metric for an integer variable, *1-pValue of Kolmogorov-Smirnov test* seems to be the safer choice. The *Euclidean distance* should only be considered when the number of subjects is large and when most integers in the range of interest were observed multiple times. Careful inspections of the cumulative imbalance distribution functions from different imbalance metrics may be particularly useful here.

Among the imbalance metrics for continuous variables, the *Area between empirical CDFs* and the *Maximum relative difference of quartiles* are the metrics of choice because of their superior correlations with other measures. It is not recommend to use the *Symmetrised Kullback-Leibler divergence* since variables with nearly zero imbalance may be highly imbalanced according to other metrics. In other words, the distributions of such a variable’s values in the two treatment groups will seem rather similar based on the *Symmetrised Kullback-Leibler divergence* but might show high differences between their means or medians, for example.

### Strengths

Thanks to the graphic representation of the (cumulative) distribution of all imbalances from a representative set of all possible allocation schemes, the *Shiny Balancer* allows the researcher deeper insight into what levels of balance are achievable for the variables in her/his study. The software tool includes both categorical and continuous variables and thus extends the scope and capabilities of “studywise minimization” [9] and “best balance” [10]. The *Shiny Balancer* also offers the possibility to experiment with different imbalance measures from which the researcher may then choose what seems best suited for her/his study’s specific needs.

Contrary to what some authors wrote [2, 9], the algorithm does not per se require that all randomisation units be enrolled in advance before the allocation can be carried out. The proposed implementation offers the possibility to take previously determined allocations into account when calculating imbalances, and can therefore be used to successively randomise subsets of units (as soon as their attributes are known). Carter et al. [8] offer some guidance to the minimum number of units to be randomised (i.e. the “block size” in a sequential blockwise allocation procedure) as well as to the minimum size of the preselected set of allocation schemes from which the final one will be chosen.

It has been argued that using covariate-constrained randomisation requires added statistical support during the allocation process [2]. However, the author feels that the intuitive design of the *Shiny Balancer* and its easy accessibility via a web link render such assistance unnecessary.

### Limitations

In its present state, the *Shiny Balancer* allocates to two treatment groups only. Weights cannot be specified for individual variables but instead for different types of variables only. In rare cases due to rounding effects, the colouring of the displayed cumulative distribution function of all imbalance values does not perfectly match the numbers below the slider. (In such cases, the numbers are correct and to be trusted over the illustration.) For the simulation part of this paper, the data sets used do not claim representativity for all conceivable study settings.

## Conclusions

The *Shiny Balancer* is an intuitive and easily accessible allocation tool that can handle both categorical and continuous variables and offers the possibility to visually explore the balancing behaviour of several well established or newly suggested imbalance metrics. The simulations have shown that for categorical variables, *χ*^2^-based imbalance measures seem to be viable alternatives to the established “quadratic imbalance” metric, and for continuous variables, the area between the empirical cumulative distribution functions or the largest difference in the three pairs of quartiles should be considered to measure imbalance. The Kullback-Leibler divergence, as proposed by Endo et al. [23], should be used with caution.

## Availability and requirements

**Project name**: Shiny Balancer

**Project home page**: http://ihamz.shinyapps.io/ShinyBalancer

**Operating system(s**): Platform independent

**Programming language**: R

**Other requirements**: Any web browser, or RStudio with libraries *shiny*, *rhandsontable*, *xlsx*, and *flexmix*

**License**: Beerware licence (revision 42)

**Any restrictions to use by non-academics**: Commercial organisations are welcome to contact the author prior to use.

## Additional file

Additional file 1: **app.R** Main application source code. **helpers.R** Definitions of imbalance measures. **simulations.R** Simulation study code. **testdataset1.xlsx** Data set 1 as Excel file. **testdataset2.xlsx** Data set 2 as Excel file. **results_bin_tds1.jpg** Scatterplots and correlations for binary/logical variable in data set 1. **results_bin_tds2.jpg** Scatterplots and correlations for binary/logical variable in data set 2. **results_cat_tds1.jpg** Scatterplots and correlations for categorical variable in data set 1. **results_cat_tds2.jpg** Scatterplots and correlations for categorical variable in data set 2. **results_int_tds1.jpg** Scatterplots and correlations for integer variable in data set 1. **results_int_tds2.jpg** Scatterplots and correlations for integer variable in data set 2. **results_con_tds1.jpg** Scatterplots and correlations for continuous variable in data set 1. **results_con_tds2.jpg** Scatterplots and correlations for continuous variable in data set 2. (ZIP 7418 KB)

## Abbreviations

1-PKS: *1-pValue of Kolmogorov-Smirnov test*
1-Pt: *1-pValue of 2sided t test*
1-PU: *1-pValue of 2sided U test*
1-PX2: *1-pValue of chi-squared test*
AbCDF: *Area between empirical CDFs*
Canb: *Canberra distance*
Eucl: *Euclidean distance*
Hell: *Hellinger distance*
Manh: *Manhattan distance*
Max: *Maximum distance*
Mrdq: *Maximum relative difference of quartiles*
SBKL: *Symmetrised Bayesian Kullback-Leibler divergence*
SKL: *Symmetrised Kullback-Leibler divergence*
X2d: *Chi-squared distance*
