# Supplementary figures and images for: The Shiny Balancer - software and imbalance criteria for optimally balanced treatment allocation in small RCTs and cRCTs
Source: BMC Med Res Methodol. 2018 Oct 16;18:108. doi: 10.1186/s12874-018-0551-5 (PMC6192202; doi:10.1186/s12874-018-0551-5)

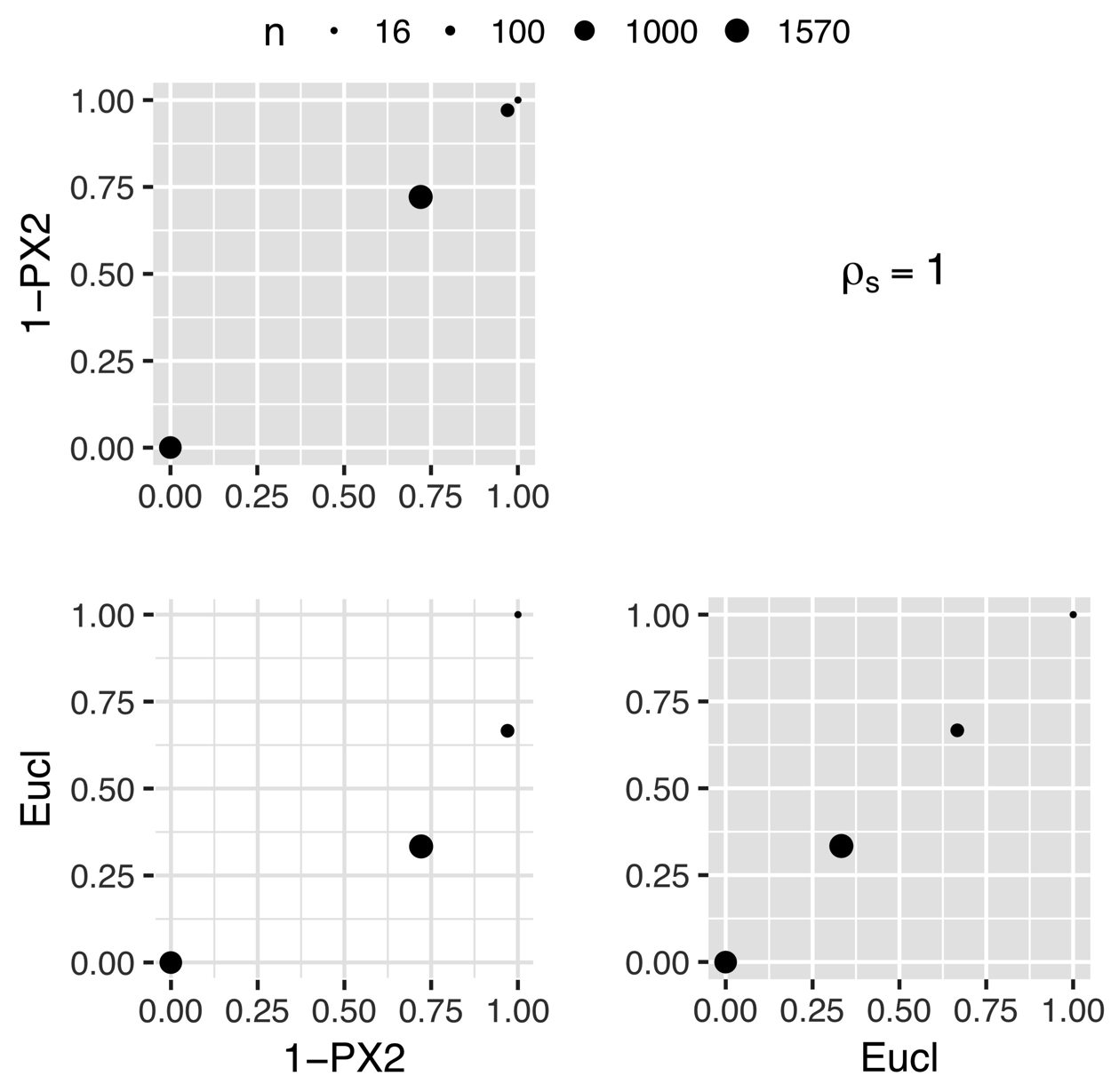

Supplement: Supplementary file 1 — app.R Main application source code. helpers.R Definitions of imbalance measures. simulations.R Simulation study code. testdataset1.xlsx Data set 1 as Excel file. testdataset2.xlsx Data set 2 as Excel file. results_bin_tds1.jpg Scatterplots and correlations for binary/logical variable in data set 1. results_bin_tds2.jpg Scatterplots and correlations for binary/logical variable in data set 2. results_cat_tds1.jpg Scatterplots and correlations for categorical variable in data set 1. results_cat_tds2.jpg Scatterplots and correlations for categorical variable in data set 2. results_int_tds1.jpg Scatterplots and correlations for integer variable in data set 1. results_int_tds2.jpg Scatterplots and correlations for integer variable in data set 2. results_con_tds1.jpg Scatterplots and correlations for continuous variable in data set 1. results_con_tds2.jpg Scatterplots and correlations for continuous variable in data set 2. (ZIP 7418 KB) [file 12874_2018_551_MOESM1_ESM.zip › results_bin_tds1R2.jpg]

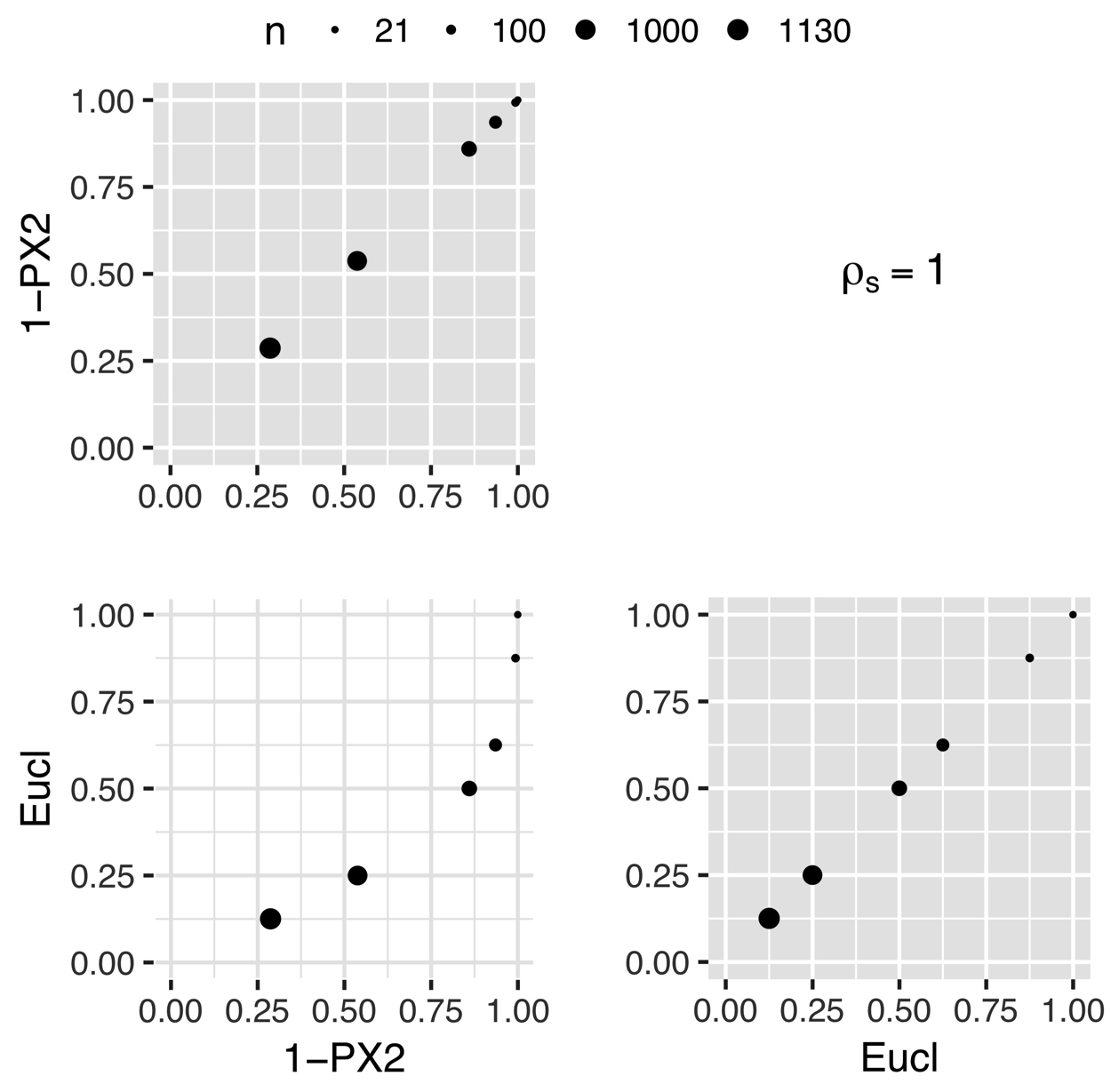

Supplement: Supplementary file 1 — app.R Main application source code. helpers.R Definitions of imbalance measures. simulations.R Simulation study code. testdataset1.xlsx Data set 1 as Excel file. testdataset2.xlsx Data set 2 as Excel file. results_bin_tds1.jpg Scatterplots and correlations for binary/logical variable in data set 1. results_bin_tds2.jpg Scatterplots and correlations for binary/logical variable in data set 2. results_cat_tds1.jpg Scatterplots and correlations for categorical variable in data set 1. results_cat_tds2.jpg Scatterplots and correlations for categorical variable in data set 2. results_int_tds1.jpg Scatterplots and correlations for integer variable in data set 1. results_int_tds2.jpg Scatterplots and correlations for integer variable in data set 2. results_con_tds1.jpg Scatterplots and correlations for continuous variable in data set 1. results_con_tds2.jpg Scatterplots and correlations for continuous variable in data set 2. (ZIP 7418 KB) [file 12874_2018_551_MOESM1_ESM.zip › results_bin_tds2R2.jpg]

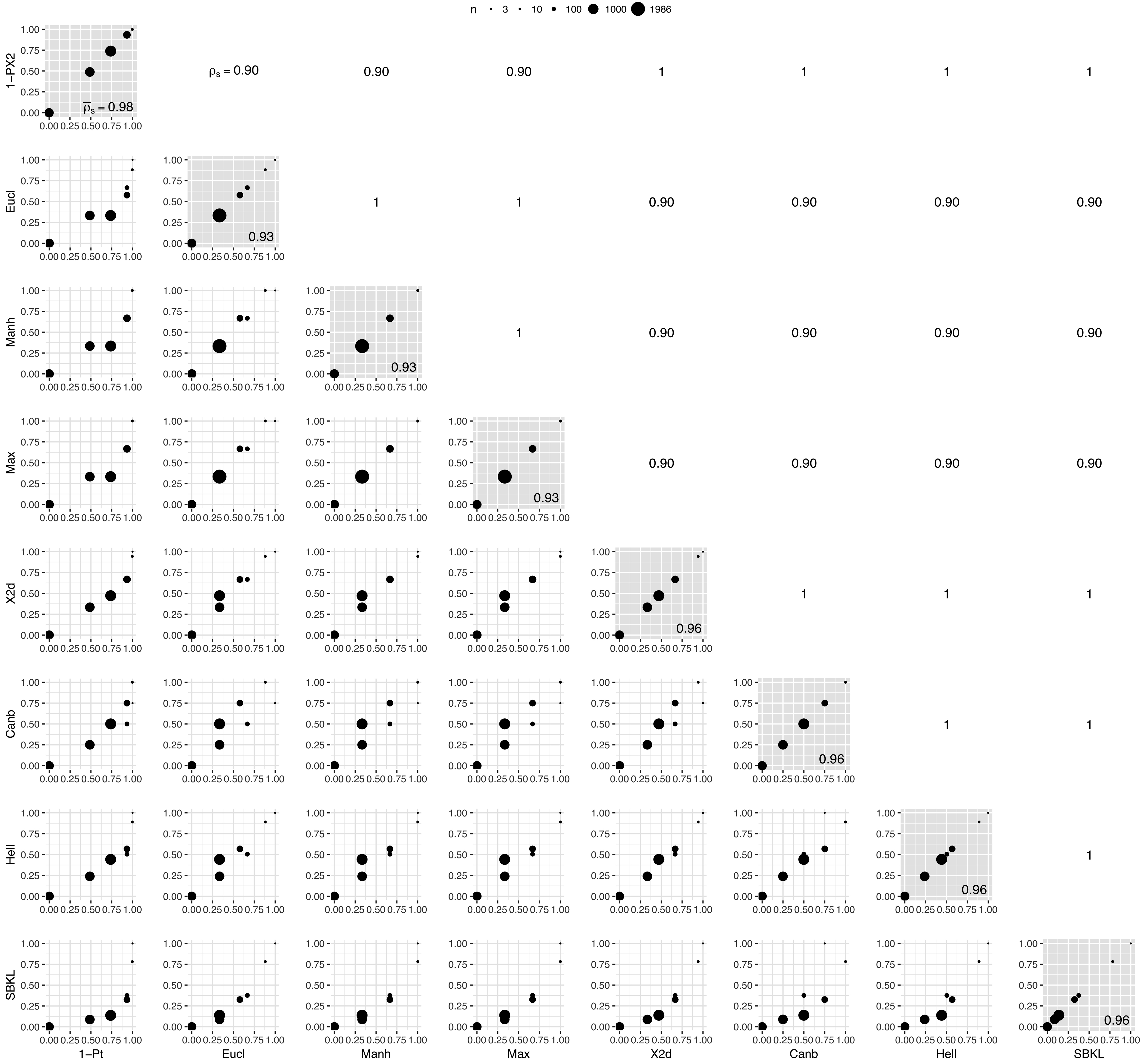

Supplement: Supplementary file 1 — app.R Main application source code. helpers.R Definitions of imbalance measures. simulations.R Simulation study code. testdataset1.xlsx Data set 1 as Excel file. testdataset2.xlsx Data set 2 as Excel file. results_bin_tds1.jpg Scatterplots and correlations for binary/logical variable in data set 1. results_bin_tds2.jpg Scatterplots and correlations for binary/logical variable in data set 2. results_cat_tds1.jpg Scatterplots and correlations for categorical variable in data set 1. results_cat_tds2.jpg Scatterplots and correlations for categorical variable in data set 2. results_int_tds1.jpg Scatterplots and correlations for integer variable in data set 1. results_int_tds2.jpg Scatterplots and correlations for integer variable in data set 2. results_con_tds1.jpg Scatterplots and correlations for continuous variable in data set 1. results_con_tds2.jpg Scatterplots and correlations for continuous variable in data set 2. (ZIP 7418 KB) [file 12874_2018_551_MOESM1_ESM.zip › results_cat_tds1R2.jpg]

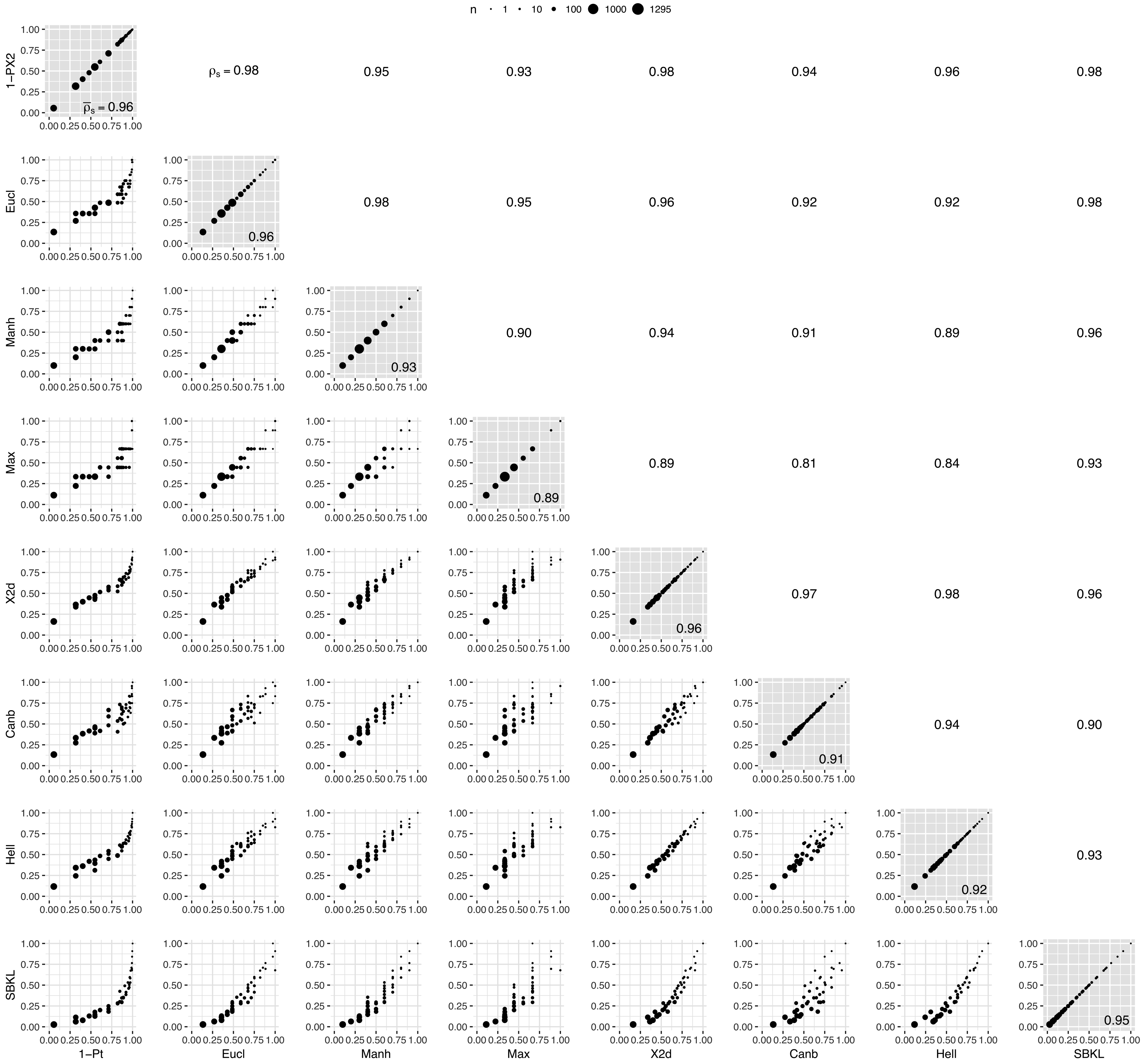

Supplement: Supplementary file 1 — app.R Main application source code. helpers.R Definitions of imbalance measures. simulations.R Simulation study code. testdataset1.xlsx Data set 1 as Excel file. testdataset2.xlsx Data set 2 as Excel file. results_bin_tds1.jpg Scatterplots and correlations for binary/logical variable in data set 1. results_bin_tds2.jpg Scatterplots and correlations for binary/logical variable in data set 2. results_cat_tds1.jpg Scatterplots and correlations for categorical variable in data set 1. results_cat_tds2.jpg Scatterplots and correlations for categorical variable in data set 2. results_int_tds1.jpg Scatterplots and correlations for integer variable in data set 1. results_int_tds2.jpg Scatterplots and correlations for integer variable in data set 2. results_con_tds1.jpg Scatterplots and correlations for continuous variable in data set 1. results_con_tds2.jpg Scatterplots and correlations for continuous variable in data set 2. (ZIP 7418 KB) [file 12874_2018_551_MOESM1_ESM.zip › results_cat_tds2R2.jpg]

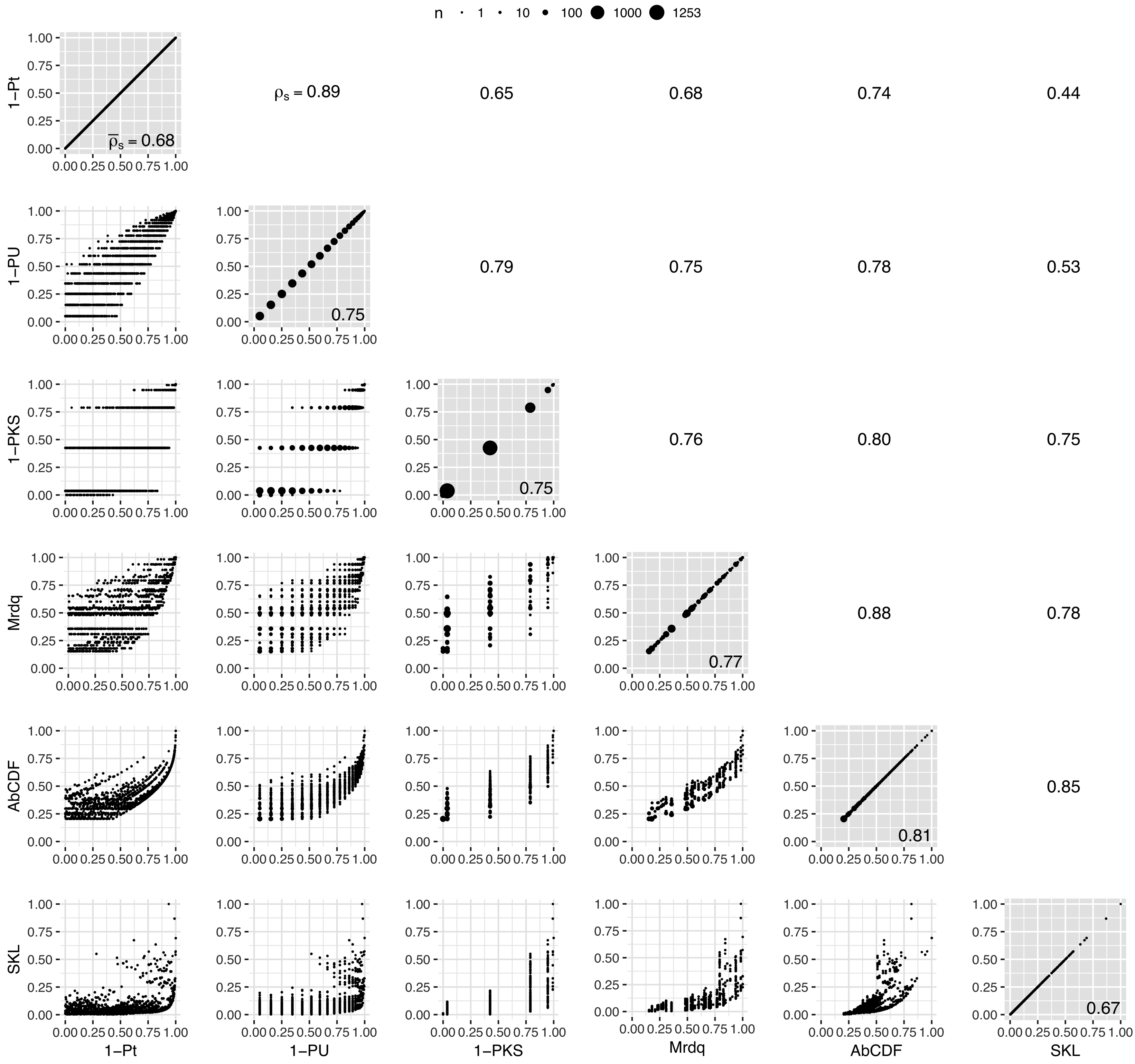

Supplement: Supplementary file 1 — app.R Main application source code. helpers.R Definitions of imbalance measures. simulations.R Simulation study code. testdataset1.xlsx Data set 1 as Excel file. testdataset2.xlsx Data set 2 as Excel file. results_bin_tds1.jpg Scatterplots and correlations for binary/logical variable in data set 1. results_bin_tds2.jpg Scatterplots and correlations for binary/logical variable in data set 2. results_cat_tds1.jpg Scatterplots and correlations for categorical variable in data set 1. results_cat_tds2.jpg Scatterplots and correlations for categorical variable in data set 2. results_int_tds1.jpg Scatterplots and correlations for integer variable in data set 1. results_int_tds2.jpg Scatterplots and correlations for integer variable in data set 2. results_con_tds1.jpg Scatterplots and correlations for continuous variable in data set 1. results_con_tds2.jpg Scatterplots and correlations for continuous variable in data set 2. (ZIP 7418 KB) [file 12874_2018_551_MOESM1_ESM.zip › results_con_tds1R2.jpg]

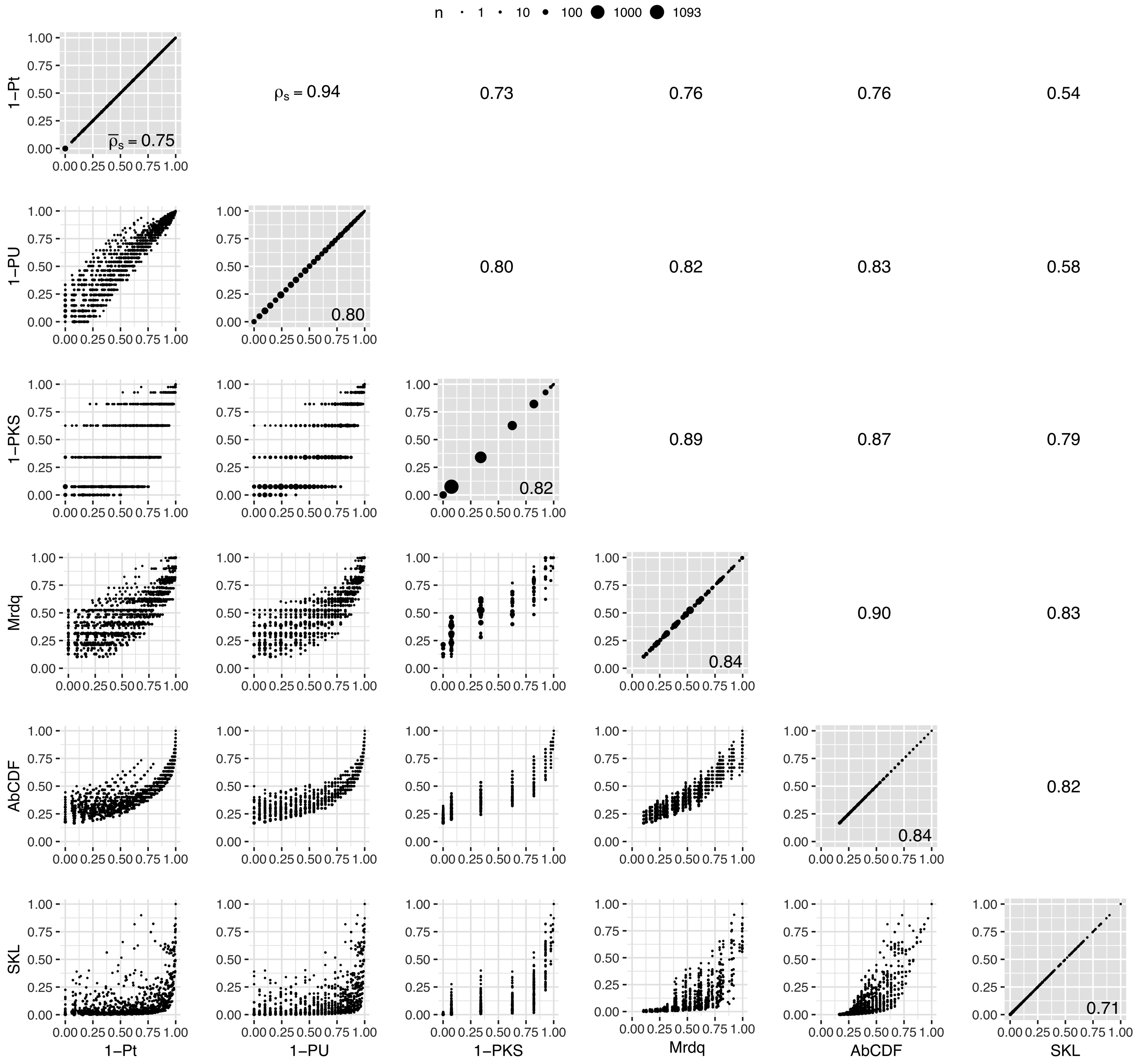

Supplement: Supplementary file 1 — app.R Main application source code. helpers.R Definitions of imbalance measures. simulations.R Simulation study code. testdataset1.xlsx Data set 1 as Excel file. testdataset2.xlsx Data set 2 as Excel file. results_bin_tds1.jpg Scatterplots and correlations for binary/logical variable in data set 1. results_bin_tds2.jpg Scatterplots and correlations for binary/logical variable in data set 2. results_cat_tds1.jpg Scatterplots and correlations for categorical variable in data set 1. results_cat_tds2.jpg Scatterplots and correlations for categorical variable in data set 2. results_int_tds1.jpg Scatterplots and correlations for integer variable in data set 1. results_int_tds2.jpg Scatterplots and correlations for integer variable in data set 2. results_con_tds1.jpg Scatterplots and correlations for continuous variable in data set 1. results_con_tds2.jpg Scatterplots and correlations for continuous variable in data set 2. (ZIP 7418 KB) [file 12874_2018_551_MOESM1_ESM.zip › results_con_tds2R2.jpg]

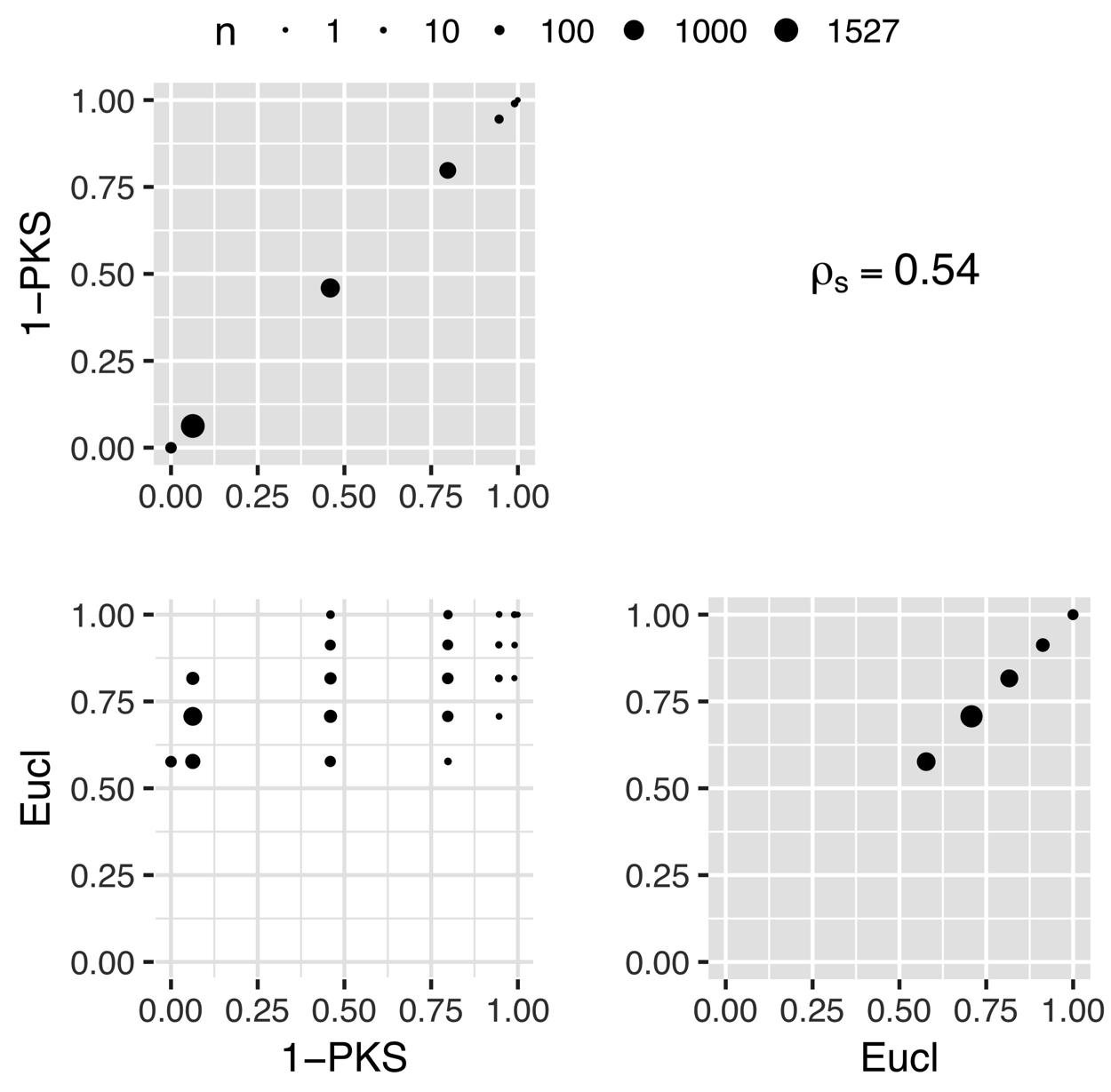

Supplement: Supplementary file 1 — app.R Main application source code. helpers.R Definitions of imbalance measures. simulations.R Simulation study code. testdataset1.xlsx Data set 1 as Excel file. testdataset2.xlsx Data set 2 as Excel file. results_bin_tds1.jpg Scatterplots and correlations for binary/logical variable in data set 1. results_bin_tds2.jpg Scatterplots and correlations for binary/logical variable in data set 2. results_cat_tds1.jpg Scatterplots and correlations for categorical variable in data set 1. results_cat_tds2.jpg Scatterplots and correlations for categorical variable in data set 2. results_int_tds1.jpg Scatterplots and correlations for integer variable in data set 1. results_int_tds2.jpg Scatterplots and correlations for integer variable in data set 2. results_con_tds1.jpg Scatterplots and correlations for continuous variable in data set 1. results_con_tds2.jpg Scatterplots and correlations for continuous variable in data set 2. (ZIP 7418 KB) [file 12874_2018_551_MOESM1_ESM.zip › results_int_tds1R2.jpg]

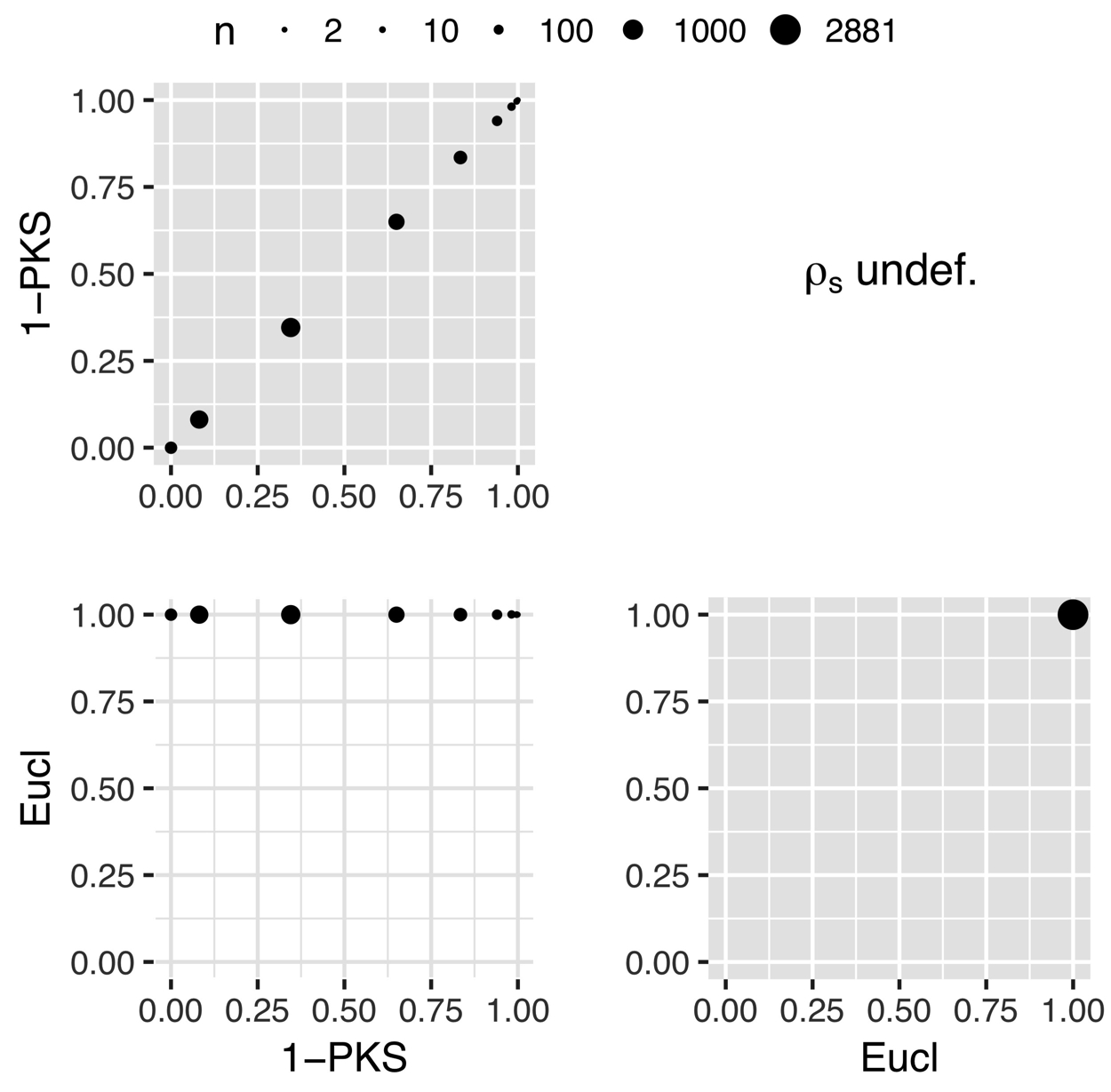

Supplement: Supplementary file 1 — app.R Main application source code. helpers.R Definitions of imbalance measures. simulations.R Simulation study code. testdataset1.xlsx Data set 1 as Excel file. testdataset2.xlsx Data set 2 as Excel file. results_bin_tds1.jpg Scatterplots and correlations for binary/logical variable in data set 1. results_bin_tds2.jpg Scatterplots and correlations for binary/logical variable in data set 2. results_cat_tds1.jpg Scatterplots and correlations for categorical variable in data set 1. results_cat_tds2.jpg Scatterplots and correlations for categorical variable in data set 2. results_int_tds1.jpg Scatterplots and correlations for integer variable in data set 1. results_int_tds2.jpg Scatterplots and correlations for integer variable in data set 2. results_con_tds1.jpg Scatterplots and correlations for continuous variable in data set 1. results_con_tds2.jpg Scatterplots and correlations for continuous variable in data set 2. (ZIP 7418 KB) [file 12874_2018_551_MOESM1_ESM.zip › results_int_tds2R2.jpg]
